# Childhood exposure to external ionising radiation and solid cancer risk

**DOI:** 10.1038/sj.bjc.6604994

**Published:** 2009-03-31

**Authors:** S Sadetzki, L Mandelzweig

**Affiliations:** 1 Cancer and Radiation Epidemiology Unit, Sheba Medical Center, Gertner Institute for Epidemiology and Health Policy Research, Tel Hashomer 52621, Israel; 2 Department of Epidemiology and Preventive Medicine, Sackler Faculty of Medicine, Tel Aviv University, Ramat Aviv, Israel

**Keywords:** ionising radiation, cancer risk, childhood exposure

## Abstract

The increasing use of ionising radiation for diagnostic purposes has raised concern about potential iatrogenic damage, especially in children. In this review, we discuss some aspects of radiation-induced cancer in relation to age at exposure and measures that should be taken for limiting exposure in this sensitive population.

Wilhelm Roentgen's discovery of X-rays in 1895, followed by Henri Becquerel's discovery of radioactivity in 1896, led to the introduction of man-made radiation. Among the important applications of these new sources of ionising radiation (IR) was their use in medical diagnosis and treatment, which spread quickly throughout the twentieth century.

Within a short time after these discoveries, reports of acute adverse effects of radiation, such as skin damage among radiation workers, began to appear. The first case of death associated with radiation-induced skin cancer was reported in 1904, while in 1911 the first case of radiation-induced leukaemia was described in a physician (Finch, 2007). Later, in the 1940s, reports of studies on mortality among physicians showed an excess of leukaemia and other cancer (attributed to exposure to IR) among radiologists and dermatologists in comparison with other physicians (Dublin and Spiegelman, 1948).

In 1913, the German Roentgen Society provided professional guidelines aimed at reducing the dangers of radiation exposure to medical workers. Among the recommendations of the British X-ray and Radium Protection Committee published several years later were a limitation of maximum work schedules, required amounts of leisure time and special accommodations for the workers (Committee for Review and Evaluation of the Medical Use Program of the Nuclear Registry Commission, 1996). The International Commission on Radiological Protection (ICRP), established in 1928, introduced important concepts such as ‘tolerance dose’, which served as an upper limit for the exposure of workers, and ‘effective dose’, which considers the overall effect of irradiation at different areas of the body (Committee for Review and Evaluation of the Medical Use Program of the Nuclear Registry Commission, 1996).

During the second half of the twentieth century, epidemiological data on radiation-induced cancer began to accumulate. Among the important sources of such data were a series of papers from the Life Span Study (LSS) cohort of atomic bomb (A-bomb) survivors. In addition, follow-up studies of cohorts treated with radiotherapy for benign conditions or cancer, as well as children exposed to lower doses because of repeated diagnostic procedures, contributed to our understanding of the dose–response relationship between exposure and risk.

In 1977, the ICRP introduced a system of dose limitations based on the principle of keeping exposures to radiation ‘As Low As is Reasonably Achievable’ (ALARA). This system included (1) justification – no practice (causing exposures of people to radiation) shall be adopted unless its introduction produces a positive net benefit (should not cause more harm than good), (2) optimisation – all exposures should be kept as low as reasonably achievable, economic and social factors being taken into account, and (3) dose limits – the dose equivalent to individuals shall not exceed the limits recommended for the appropriate circumstances (US Department of Energy, 1997).

## Childhood exposure to IR

Specific concern over the potentially harmful effects of exposure to radiation in children began to develop in the 1940s when it was suggested that fluoroscopy use in infants should be restricted according to clinical indications, and that attention should be given to calibrating the machines to limit the exposure (Buschke and Parker, 1942). In the 1950s, Alice Stewart proposed that exposure to weak irradiation could initiate malignant changes in a foetus or very young child. This hypothesis was based on the findings of a case–control study which showed that almost twice as many mothers of children who died from leukaemia or malignant disease before the age of 10 had undergone X-ray examinations of the abdomen during pregnancy (pelvimetry) in comparison with mothers of controls (Stewart *et al*, 1956). Later analyses showed that this excess risk was inversely related to foetal age and increased with the number of films taken (Bithell and Stewart, 1975).

An increased susceptibility of children to radiation-induced cancer is biologically plausible because of the fact that their tissues are still growing and therefore the dividing cells are more prone to somatic genetic damage. In addition, children have a longer life expectancy during which oncogenic effects may develop (Brody *et al*, 2007; Shah and Platt, 2008).

The purpose of this review is to summarise data on the relationship between childhood exposures to external IR and cancer risk, with specific attention to the need for adequate measures for limiting the risk of exposure in this sensitive population.

## Association between age at exposure and risk

Estimates of excess risk associated with exposure to IR are generally presented in terms of excess relative risk per gray (ERR per Gy) and excess absolute risk (EAR per Gy). The ERR associated with an exposure represents the ratio of the difference between the rate of the disease in the exposed and unexposed groups to the rate in the unexposed group. The ERR per dose is defined on the basis of the assumption that the ERR is proportional to the dose and represents the ERR for a unit dose of radiation. Excess absolute risk (EAR) per dose refers to the additional risk that a radiation dose contributes beyond the background risk.

A recent publication from the LSS on risk of solid cancer (malignancies that form tumours in solid organs, as opposed to leukaemia, which affects the blood) among atomic bomb survivors exposed to radiation doses ranging from <0.005 to 4 Gy included data showing the effect of age at exposure on excess risk for all solid cancer and by specific sites (Preston *et al*, 2007). For all solid cancer, the ERR per Gy decreased with increasing age at exposure, with estimates at attained age 70 of 0.72 (90% CI: 0.52–0.98), 0.64 (90% CI: 0.51–0.79), 0.41 (90% CI: 0.33–0.50), and 0.41 (90% CI: 0.29–0.53) for survivors aged 0–9, 10–19, 20–39, and 40+, respectively, at the time of the bombings. The EARs per 10^4^ PY of solid cancer at attained age 70 in the LSS were also higher for those who were younger at the time of exposure, with rates of 90 (90% CI: 68–113), 52 (90% CI: 43–60), and 30 (90% CI: 22–39) for ages 10, 30, and 50, respectively.

**Figure 1:**
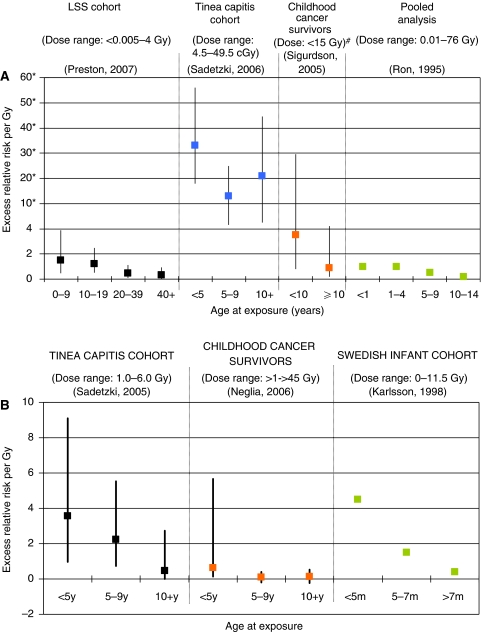
(**A**) Excess relative risk of thyroid cancer by age at exposure among selected cohorts exposed to ionising radiation. Dose ranges refer to doses to the thyroid gland. (**B**) Excess relative risk of brain cancer by age at exposure among selected cohorts treated with radiotherapy. Dose ranges refer to doses to the brain. ^*^Please note the change in the scale of the *y*-axis. #ERR estimates presented here are for survivors aged <10 and ⩾10 years who were exposed to low doses.

The pattern of decreasing risk with increasing age at exposure was particularly striking for thyroid cancer (Preston *et al*, 2007). This inverse association is supported by data from cohorts exposed to radiotherapy for cancer during childhood (Sigurdson *et al*, 2005), tinea capitis (Sadetzki *et al*, 2006), and other benign conditions, as shown in Figure 1A. The results of a pooled analysis, including individuals from six studies who were under the age of 15 at the time of radiation exposure, yielded an ERR per Gy of 7.7 (95% CI: 2.1–28.7) and an EAR per 10^4^ PY Gy of 4.4 (95% CI: 1.9–10.1) for risk of thyroid cancer. A trend for decreasing risk with increasing age at irradiation was observed (Ron *et al*, 1995). The investigators suggested that these findings might indicate a greater radiation effect during periods of rapid cell proliferation, which occur when the thyroid gland is developing.

For many years, brain tissue was considered to be resistant to radiation damage. Four studies that have quantified the risk between IR and brain cancer have reported estimations that vary in terms of the magnitude of the risk per Gy. The Israeli tinea capitis study found an ERR per Gy of 1.98 (95% CI: 0.73–4.69) for glioma after a mean dose to the brain of 1.5 Gy (range: 1.0–6.0 Gy) (Sadetzki *et al*, 2005); a pooled analysis of two Swedish cohorts of infants who received radium treatment for haemangiomas (mean absorbed intracranial dose: 7 cGy; range: 0–11.5 Gy) estimated the ERR per Gy for all brain tumours as 2.7 (95% CI: 1.0–5.6) (Karlsson *et al*, 1998); a childhood cancer survivor study reported an ERR per Gy of 0.33 (95% CI: 0.07–1.71) for glioma (Neglia *et al*, 2006) after radiation doses ranging from <1 Gy to >45 Gy; and the LSS found an ERR per Gy for glioma of 0.6 (95% CI: −0.2 to 2.0) (Preston *et al*, 2002b). Evidence of increasing risk of brain cancer with younger age at exposure has been provided by the first three aforementioned studies (Figure 1B), whereas for the LSS, the risk was non-significantly higher for those exposed before age 20. It is interesting to note that even among infants, the risk of those treated for haemangioma when they were <5 months old was much greater than that of those treated over the age of 5 months (Figure 1B).

Radiation effects on female breast cancer rates have been studied extensively. The results of pooling eight cohorts showed that age at exposure is an important modifier of the breast cancer ERRs with higher risks for younger ages, so that exposure after age 50 carries a lower risk than exposure earlier in life. No simple unified summary model adequately described the excess risks in all populations, suggesting that host factors (e.g. earlier benign breast disease or reproductive factors) are determinants of the variations in the ERR with age at exposure (Preston *et al*, 2002a).

Among Canadian women who were examined by fluoroscopy during treatment for tuberculosis (range of radiation dose: <0.01–⩾10 Sv), the ERR per Sv for breast cancer mortality was shown to decrease with increasing age at exposure (*P*=0.0003) (Howe and McLaughlin, 1996). In the latest results of the LSS, a statistically significant age-at-exposure effect of −19% per decade increase in ERR (90% CI: −33 to −4%) was noted for breast cancer. However, the addition of attained age to the model improved the fit significantly and reduced the age-at-exposure effect estimate to 0, whereas both attained age and age at exposure had a significant joint effect on the EAR (Preston *et al*, 2007). Differences between studies still remain regarding the influence of age at exposure around menarche and menopause on the risk.

A strong inverse association between age at exposure and risk has also been noted for non-melanoma skin cancer (*P*<0.001) in the LSS (Preston *et al*, 2007). This finding is supported by data from the tinea capitis study, in which the fitted relative risk for basal cell carcinoma decreased from 19.0 (95% CI: 14.4–28.4) for irradiation at age ⩽4, to 5.9 (95% CI: 3.3–11.0) for exposure at age 5–9, and 2.0 (95% CI: 1.3–4.0) among those exposed at ages 10–15 (Ron *et al*, 1991).

It is interesting to note that for lung cancer a different picture was observed, in which the ERR increased with increasing age at exposure. This finding may be attributed to the additive joint effects of radiation and smoking (Pierce *et al*, 2003; Preston *et al*, 2007).

The evaluation of childhood cancer risk in the LSS study was limited by the fact that the tumour registries were only established in 1958, and therefore, data on cancer incidence during the first 13 years after the bombings were unavailable. An assessment of risk among survivors diagnosed during adolescence identified eight cases of solid cancers between the ages of 13 and 19, yielding an ERR per Gy of 19.8 (90% CI: 6–77) for age<20 years. It has been suggested that if follow-up of the cohort had been initiated at an earlier stage, the risks associated with childhood cancer would have been extremely high (Preston *et al*, 2007).

## Age at exposure and temporal patterns of risk (attained age and latency period)

Age at exposure, time since exposure, and attained age are collinear variables, and their combined effect differs from the individual effects of each factor. Thus, the association between these factors and radiation-induced cancer risk can only be estimated for two of the three at any one time, and the true causal effect of age at exposure on radiation-induced cancer cannot be assessed (Lagarde, 2006).

Analyses of the LSS provided evidence of a decrease in ERRs with increasing attained age, for any age at exposure. The age-at-exposure effect is shown by a 20% decrease in attained-age-specific ERRs per decade increase in age at exposure. In contrast, EARs were shown to increase throughout life regardless of the age at exposure, and there have been indications that for solid cancers, attained-age-specific EARs are higher for those exposed at younger ages (Preston *et al*, 2003).

## Reducing exposure to IR in children

A profound discussion on the dose–response association is beyond the scope of this review. Scientific consensus groups as well as regulatory agencies have endorsed the use of the linear, no-threshold model in determining standards of protection from the risk of solid cancer associated with exposure to IR at low dose levels (Committee for Review and Evaluation of the Medical Use Program of the Nuclear Registry Commission, 1996). The data described above highlight the need for applying the ALARA principle for reducing exposure to IR, with specific attention to children, who represent a subgroup with increased radiosensitivity. The importance of this approach has increased as a result of the growing use of diagnostic procedures that usually involve low-dose radiation, and specifically for computed tomography (CT) scanning, which commonly involves 30–90 mSv per 2–3 scans. This dose is comparable to the low doses of radiation (5–150 mSv), to which a subgroup of 25 000 A-bomb survivors was exposed. The statistically significant increase in risk of solid cancer associated with the latter doses suggests that the organ doses corresponding to a CT study may also increase the risk of cancer (Brenner and Hall, 2007).

It should also be noted that a dramatic increase in the use of CT over time has been observed, as indicated by estimates of 2 million per year in the United States in 1980 rising to 69 million in 2007 (Hall and Brenner, 2008). Data from the United States and Europe have shown that although CT constitutes only 5–10% of all imaging procedures, 40–67% of all exposure to medical diagnostic radiation can be attributed to this procedure (Sadetzki, 2007).

An important development in the attempt to reduce unnecessary exposure of children to IR was the recent establishment of the Alliance for Radiation Safety in Pediatric Imaging. This coalition, which comprises members of leading medical societies, agencies and regulatory groups, aims to influence patient care and change practice through an educational and awareness campaign entitled ‘Image Gently’. The campaign includes a four-point strategy regarding performing pediatric CT scans, including the need to reduce or ‘child-size’ the amount of radiation used, scan only when necessary, scan only the indicated region, and refrain from multiphase scanning unless it is necessary (Goske *et al*, 2008).

Other strategies aimed at protecting pediatric patients include consideration of alternatives to CT, such as ultrasound and magnetic resonance imaging, and shielding of the breast areas (including the nipples and breast buds in young girls), thyroid, and gonads when possible during CT scans (Shah and Platt, 2008).

The need to increase the awareness of professionals regarding the late health effects of exposure to IR has been shown in a study of radiography conducted in five Israeli neonatal care units, which identified unnecessary overexposure to IR in a substantial proportion of body regions beyond those that were ordered. This problem was particularly prevalent in radiographs of the abdomen, in which the neck and upper chest were exposed in 45 and 64%, respectively, which did not comply with international recommendations. Other problems encountered included the need to repeat up to 20% of the radiographs, thereby increasing the overall exposure of the infant to radiation, and the lack of shielding of the gonads in most of the X-rays taken. Correction of this situation does not require investment of resources. By simply raising awareness among the staff with better guidelines regarding exposure to IR, these issues can be resolved (Bader *et al*, 2007).

Another measure that has been proposed for limiting exposure to diagnostic radiation in children is the incorporation of personal information on lifetime IR exposure in computerised medical systems. Such information could include the numbers and types of procedures carried out, the ages at which they were carried out, and calculations of cumulative doses absorbed by different organs. Consideration of these factors could be important in determining to which diagnostic examinations the patient should be referred (Sadetzki, 2007).

Thus, there are also legal and ethical implications of exposure to diagnostic IR that need to be addressed, such as the right of a parent to be informed of the risks involved in the procedures to which his or her child has been referred.

## Conclusion

The use of IR in medicine has provided a wide range of possibilities for diagnosis and treatment. Constant refinement of the techniques used in radiological examinations has produced greater resolution in imaging, facilitating greater accuracy of assessments. However, along with the important benefits of medical applications of IR, there are risks that need to be considered as well. Children constitute a subgroup at greater risk from exposure to radiation because of their greater degree of radiosensitivity and because of the many years of life ahead of them during which they could develop radiation-induced cancer. In order to minimise the risks, it is essential that procedures involving low doses of IR be used with discretion, in a responsible way. Although the risks involved may be relatively small, the ancient concept expressed by Hippocrates of ‘*Primum non nocere’* (to help, or at least to do no harm) should serve as a guide when assessing the appropriateness of radiation-based imaging procedures in pediatric patients.
